# EXPOsOMICS: final policy workshop and stakeholder consultation

**DOI:** 10.1186/s12889-018-5160-z

**Published:** 2018-02-15

**Authors:** Michelle C. Turner, Paolo Vineis, Eduardo Seleiro, Michaela Dijmarescu, David Balshaw, Roberto Bertollini, Marc Chadeau-Hyam, Timothy Gant, John Gulliver, Ayoung Jeong, Soterios Kyrtopoulos, Marco Martuzzi, Gary W. Miller, Timothy Nawrot, Mark Nieuwenhuijsen, David H. Phillips, Nicole Probst-Hensch, Jonathan Samet, Roel Vermeulen, Jelle Vlaanderen, Martine Vrijheid, Christopher Wild, Manolis Kogevinas, on behalf of the EXPOsOMICS Consortium

**Affiliations:** 1 Barcelona Institute for Global Health (ISGlobal), Barcelona, Spain; 2 Universitat Pompeu Fabra (UPF), Barcelona, Spain; 3 CIBER Epidemiología y Salud Pública (CIBERESP), Madrid, Spain; 4 McLaughlin Centre for Population Health Risk Assessment, University of Ottawa, Ottawa, Canada; 5 MRC-PHE Centre for Environment and Health, School of Public Health, Imperial College London, Norfolk Place, W2 1PG, London, UK; 6 International Agency for Research on Cancer, Lyon, France; 7 National Institute of Environmental Health Sciences, Research Triangle Park, Durham, North Carolina USA; 8 Former WHO Chief Scientist and Representative to the European Union, Brussels, Belgium; 9 Public Health England, London, UK; 10 University of Basel, Swiss Tropical and Public Health Institute, Basel, Switzerland; 11 National Hellenic Research Association, Athens, Greece; 12 World Health Organisation, Bonn, Germany; 13 Emory University, Atlanta, USA; 14 University of Hasselt, Hasselt, Belgium; 15 King’s College London, London, UK; 16 Colorado School of Public Health, Aurora, USA; 17 Utrecht University, Utrecht, Netherlands; 18 IMIM (Hospital del Mar Medical Research Institute), Barcelona, Spain

**Keywords:** Exposome, Workshop report, External exposome, Internal exposome, Air pollution, Water contamination, Adductomics, Statistics, Policy

## Abstract

The final meeting of the EXPOsOMICS project “Final Policy Workshop and Stakeholder Consultation” took place 28–29 March 2017 to present the main results of the project and discuss their implications both for future research and for regulatory and policy activities. This paper summarizes presentations and discussions at the meeting related with the main results and advances in exposome research achieved through the EXPOsOMICS project; on other parallel research initiatives on the study of the exposome in Europe and in the United States and their complementarity to EXPOsOMICS; lessons learned from these early studies on the exposome and how they may shape the future of research on environmental exposure assessment; and finally the broader implications of exposome research for risk assessment and policy development on environmental exposures. The main results of EXPOsOMICS in relation to studies of the external exposome and internal exposome in relation to both air pollution and water contaminants were presented as well as new technologies for environmental health research (adductomics) and advances in statistical methods. Although exposome research strengthens the scientific basis for policy development, there is a need in terms of showing added value for public health to: improve communication of research results to non-scientific audiences; target research to the broader landscape of societal challenges; and draw applicable conclusions. Priorities for future work include the development and standardization of methodologies and technologies for assessing the external and internal exposome, improved data sharing and integration, and the demonstration of the added value of exposome science over conventional approaches in answering priority policy questions.

## Background and objectives

The final meeting of the EXPOsOMICS project “Final Policy Workshop and Stakeholder Consultation” took place in Brussels, Belgium on 28–29 March 2017. The meeting programme was structured around the main research topics in EXPOsOMICS, and the presentations fell into four main themes: presentation of the main results and the advances in exposome research achieved through the EXPOsOMICS project; presentations on other parallel research initiatives on the study of the exposome in Europe and in the United States and their complementarity to EXPOsOMICS; lessons learned from these early studies on the exposome and how they may shape future research on environmental exposure assessment; and finally the broader implications of exposome research for hazard identification, risk assessment and policy development on environmental exposures. In addition, the programme included three plenary sessions – on the external exposome, the internal exposome and on policy translation – each of which was led by two discussants who introduced the themes and promoted discussion amongst the participants. This paper summarizes the presentations and discussions at this meeting.

## Overview of EXPOsOMICS: Relevance to hazard identification and risk assessment

There are two broad interpretations of the exposome concept, or the totality of environmental exposures from conception onwards, and they are complementary [1–3]. One, called “top-down”, is mainly interested in identifying new causes of disease by an *agnostic* approach based on omic technologies. This first approach is sometimes called “EWAS”, or “exposome-wide association study”, and utilizes methods such as metabolomics or adductomics to generate new hypotheses on disease etiology. The second general approach is called “bottom-up” and starts with a set of exposures or environmental compartments to determine the pathways or networks by which such exposures lead to disease, i.e. which pathways/networks are perturbed. We have used the latter approach in the EXPOsOMICS investigation as we explain below [4].

The context of EXPOsOMICS is the rapidly developing exposome field, including exposure assessment and the use of omic technologies. The overall objective of the EXPOsOMICS project was to comprehensively integrate both external and internal exposomes at the individual level and to provide a holistic approach to exposure science. The scientific questions that were addressed by EXPOsOMICS are presented in Table 1. The main results of EXPOsOMICS in relation to studies of the external and internal exposome are presented below as well as those for new technologies for environmental health research (adductomics) and advances in statistical methods (Table 1). EXPOsOMICS focussed on two high priority environmental pollutants, air pollution and water contaminants, exploiting existing short-term and long-term European population studies, integrating findings using harmonized external and internal assessment approaches in studies of critical life stages (conception through old age) to explore associations over the life-course [4].

**Table 1:** Summary of research questions and main outputs of the EXPOsOMICS project

Research Question	Main Outputs
(1) Is it possible to refine exposure assessment to air pollution and water contaminants using a combination of personal exposure monitoring and omic technologies?	-Detailed 24 h PEMs for PM_2.5_ and UFP conducted on 200 participants -LUR models for PM2.5 and NO2 developed for Western Europe -LUR models for UFP and oxidative potential of PM2.5 developed in six European areas -Short-term exposure to air pollutants induced changes in omic profiles, including gene expression, metabolites, and immune markers -A expanded range of DBPs in air, water and/or in biological samples such as exhaled breath and urine were measured/modeled -Numerous metabolic and transcriptional changes due to swimming in a chlorinated pool
(2) Will that refinement lead to more accurate estimates of the association with selected diseases, by reducing measurement error?	-Increased RRs for total mortality and ischemic heart disease and asthma incidence using PM_2.5_ deattenuation factors from the PEM study -Positive association between high levels of brominated THM exposure and colorectal cancer risk
(3) Do new approaches allow the investigation of the effects of mixtures in addition to single components?	-Lack of overlap between omics signals for different air pollutants (may suggest the ability of omics to detect pollutant-specific biological effects)
(4) Do they improve the investigation of dose-response relationships?	-Omics signals occurred at very low levels of exposure and following short-term exposure
(5) Is it possible to strengthen causal reasoning by using the “meet-in-the-middle” concept, i.e. investigate the temporal sequence of exposure, biological pathway perturbation and disease onset?	-Metabolomics was used to study meet-in-the-middle pathways linking air pollution to adult-onset asthma with observed evidence of the involvement of both linoleate metabolism and carnitine pathways lending causal credibility to the association -Transcriptional and microRNA changes observed after swimming were linked to bladder and colon cancer risk from previous studies
(6) Is it possible to use the exposome approach to study the life-course epidemiology of environmental diseases?	-Air pollution impacts on both asthma and cardiovascular disease via pro-inflammatory and oxidative stress pathways, consistent with accumulation of oxidative molecular damage over years of exposure - Known candidate transcriptome profiles of blood pressure/insulin in adulthood were associated with prenatal PM exposure at birth -Longitudinal air pollution exposures were associated with alterations in genes involved in neurotransmission and tumor suppression pathways

*Abbreviations*: *DBP* Disinfection by-product, *LUR* Land-use regression, *NO*_*2*_ Nitrogen dioxide, *PM*_*2.5*_ Particulate matter less than 2.5 μm in diameter, *PEM* Personal exposure measurements, *RR* Relative risk, THM Trihalomethane, *UFP* Ultrafine particles

## Air pollution

### External Exposome

John Gulliver presented advances in monitoring of personal exposures to air pollution in EXPOsOMICS. Main goals of the project were to develop new land use regression (LUR) models for ultrafine particles (UFP) and oxidative potential, as well as undertake personal measurements of particulate matter less than 2.5 μm in diameter (PM2.5) and UFP to investigate the potential for exposure misclassification in using outdoor models of exposure at the residential address as the sole means of exposure assessment. Detailed 24 h PEM for particulate matter less than 2.5 μm in diameter (PM_2.5_) and ultrafine particle (UFP) estimates were conducted on approximately 200 participants with data on individual positioning and accelerometry from smartphones in four European countries. Findings contrasting personal UFP exposures for individuals living near to traffic and in background locations suggested that other microenvironments (journeys, work, home indoors etc.) are important contributors in determining levels of personal UFP exposure. LUR models for PM_2.5_ and nitrogen dioxide (NO_2_) concentrations in Western Europe were developed for harmonised exposure assessment in epidemiological studies combining either satellite data with local predictors (PM_2.5_) or chemical transport modelling data with local predictors (NO2) [5]. New outdoor spatial LUR models were also developed for UFP in six European areas with good performance in predicting 24 h outdoor home exposures [6] as well as for oxidative potential of PM_2.5_ in five European areas that provided an independent exposure metric that was not strongly correlated with PM_2.5_ concentrations. Results provide insight into the contribution of different microenvironments in air pollution exposure and new exposure models for use in epidemiological studies. Future efforts include the use of PEM technology in larger study populations and epidemiological studies.

Nicole Probst-Hensch presented the contribution of the exposome approach towards understanding the association of long-term PM_2.5_ exposure and asthma/cardiovascular disease (CVD) in adults (see also below). Relative risk (RR) estimates from the ESCAPE study for total mortality, ischemic heart disease (IHD) incidence, and asthma incidence [7, 8] were calibrated using low-end and high-end deattenuation factors for PM_2.5_ exposure obtained from the PEM study (above). Point risk estimates were increased in comparison with non-calibrated estimates based on LUR alone. Estimates of attributable burden of IHD and asthma incidence due to PM_2.5_ exposure in the EU-28 and Switzerland were also presented, with an approximate 1.5 to 2-fold increase in disease burden obtained using calibrated RR estimates from ESCAPE. Results suggest that the contribution of PM_2.5_ to the asthma and CVD burden may be underestimated using existing methods.

### Internal Exposome

Soterious Kyrtopoulos discussed the identification of biomarkers of exposure in short-term experimental studies and of associated metabolic pathways potentially linked with health risks. In initial results from the Oxford Street study [9], a randomized cross-over study of 59 adults invited to walk for 2 h in both a high (Oxford Street) and a low (Hyde Park) air pollution setting, short-term exposure (2 h) to air pollutants induced changes in omic profiles, especially gene expression and metabolites which differed among the different air pollutants with little overlap. In pathway/network analysis, there was evidence of links with energy metabolism and inflammation pathways, including the carnitine shuttle pathway which was most significantly affected for NO_2_. For most compounds, levels returned to pre-exposure levels by 24 h. For transcriptomics, the greatest number of signals was also related with NO_2_ exposure with little overlap between pollutants which in pathway analysis were related with the immune system and platelet aggregation. Cross-omic analysis investigated overlap between carnitine associated genes and transcriptomics markers.

Roel Vermeulen and Jelle Vlaanderen provided an overview of the identification of omics markers of short-term exposure to air pollution and the potential for using PEM data to improve exposure estimates in larger, long-term studies of air pollution. Although long-term air pollution concentrations have been associated with changes in omics markers, these studies are limited by inconsistent findings across studies, crude exposure assessment, and limited samples per individual [10–12]. The link with acute health effects is also often unclear. Based on the PEMs of PM_2.5_ and UFP in four European countries (see above), with three exposure and two omics measurements per individual, positive associations were observed between different immune markers and personal PM_2.5_ and UFP concentrations. There were also several CpG sites that were significantly associated with personal PM_2.5_ concentrations. Findings based on repeated measurements provide knowledge around how exposure and omics markers vary within individuals to be able to better assess patterns in omics markers and health outcomes between individuals. Further work to contrast findings with those from studies of long-term exposure is needed. Once omics signals strongly associated to specific air pollutants have been identified, these might provide an avenue for calibrating air pollution exposure estimates in cohort studies via regression calibration techniques [13], potentially reflecting a more biologically relevant exposure metric.

Nicole Probst-Hensch further presented the contribution of the exposome approach towards understanding the association between long-term exposure to air pollution and asthma/CVD in adults (see also above). Results from mediation analyses were consistent with air pollution impacting on both asthma and CVD via pro-inflammatory and oxidative stress pathways, albeit with different molecules involved – consistent with accumulation of oxidative molecular damage over years of exposure. Ayoung Jeong used metabolomics to study meet-in-the-middle pathways linking air pollution to adult-onset asthma in the SAPALDIA and EPIC cohorts, with observed evidence of the involvement of both linoleate metabolism and carnitine pathways.

Lastly Tim Nawrot described multi-omic analyses to identify signals associated with in utero exposures to air pollution (particulate matter less than 10 μm in diameter (PM_10_) and PM_2.5_) and early life effects in five European birth cohorts. Transcriptome wide microarray analysis was undertaken to understand biological and developmental origins of health and disease mechanisms related with blood pressure and insulin; epigenome-wide analysis to study longitudinal signals associated with early life exposure; and metabolome-wide analysis of cord blood to investigate metabolic signatures of birthweight and the influence of PM exposure. Known candidate transcriptome profiles of blood pressure/insulin in adulthood [14] were associated with prenatal PM exposure at birth with different responses observed in boys and girls. The top significant newborn PM transcripts observed have functional consequences based on associations with cord metabolites and protein targets. Longitudinal air pollution exposures were associated with alterations in genes involved in neurotransmission and tumor suppression pathways. Further work to examine consistency in findings across studies and across omics platforms is needed.

## Water contamination

### External Exposome

Manolis Kogevinas presented work that focused on chemical contaminants in water produced during the process of disinfection. Hundreds of disinfection by-products (DBPs) are produced when disinfecting water usually through chlorination [15]. Some of them are animal carcinogens, several are mutagens or have shown genotoxicity in a variety of assays, and epidemiological studies have associated exposure to trihalomethanes (the most common DBPs) with bladder cancer [16]. There is only limited evidence on the association with colorectal cancer in humans [17]. In EXPOsOMICS, a short-term study (PISCINA2) measured an expanded range of DBPs (trihalomethanes, haloacetic acids, MX, chloramines, haloacetonitriles) in air, water and/or in biological samples such as exhaled breath (e.g. trihalomethanes) and urine (haloacetic acids) from study subjects, overcoming traditional approaches that measure only trihalomethanes [18]. In mother-child cohorts, external exposure measurement included determinations of a range of DBP chemicals in drinking water (trihalomethanes (THM), haloacetic acids, haloacetonitrile), with some available from the EU-funded HiWate project [18]. In the colorectal cancer study (MCC-Spain), exposure modeling of DBPs was based on the evaluation of lifetime residential history together with the collection of historical information on DBPs in the relevant regions and water toxicity testing from short-term studies. Results did not show an overall association of colorectal cancer with THM exposure [17]. An increase in risk was observed only for subjects exposed to high levels of brominated THM concentrations; experimental evidence indicates that brominated compounds are more toxic than chlorinated compounds [19, 20]. Finally, although THMs are regulated in the EU there are no central statistics available on THM exposure in EU countries. As part of the burden of disease component of EXPOsOMICS, regulatory or other agencies in all EU countries were contacted and a map with average current THM levels in the 28 EU countries was produced for the first time. The map showed considerable differences between countries with very low levels observed in countries such as Denmark and The Netherlands and high levels observed in countries such as Spain, Romania and Ireland. A burden of disease estimation indicated that more than 6000 bladder cancer cases can be attributed to THM exposure in the EU each year.

### Internal Exposome

Part of the controversy concerning potential health effects from exposure to chemical contaminants produced during disinfection i.e. chloroform or bromoform, is due to the fairly low toxicity of these chemicals at the concentrations usually observed in drinking water [20]. Manolis Kogevinas presented work conducted in EXPOsOMICS in a semi-experimental study (PISCINA2) in swimmers in an indoor pool evaluating short term effects and in a population-based study evaluating long-term exposures and showed that numerous hits were identified when contrasting pre- and post-swimming omic profiles. Transcriptomics, targeted proteomics and metabolomics were examined in the swimming pool study together with an evaluation of genotoxicity biomarkers (micronuclei) and markers of lung epithelium permeability (club cell protein – CC16). The study on proteomics indicated that swimming in a chlorinated pool induces perturbations of the immune response through acute alterations of patterns of cytokine and chemokine secretion [21]. The transcriptomics analysis identified more than 1700 genes and several microRNAs that were significantly associated with exposure to at least one DBP. Among transcripts that had not been previously reported to be associated with physical activity, a large number of hits remained associated with DBP exposure and several of those are linked with bladder and colon cancer. Numerous molecular changes were identified in the metabolomic analysis following the swimming experiment and related to exposure changes. No clear associations were observed for the genotoxicity biomarkers and this contrasted with an earlier swimming pool study that was conducted among swimmers with very high levels of brominated compounds [22]. Results for metabolomics, proteomics and methylation following long-term exposure in the MCC-Spain study were also presented. Numerous hits were identified although overall findings were less prominent than those observed in the short-term swimming pool study. Some proteomic markers (mainly interleukins) were detected in association with long-term THM exposure from the MCC study controls.

## Methods

### New Technologies for Environmental Health Research – Adductomics

David Phillips presented “New technologies for environmental health research – adductomics”. Adductomics, the untargeted detection of DNA or protein adducts of endogenous or exogenous origin, is a new field in exposome research. Many of the studies in this area focus on the untargeted analysis of protein adducts in human serum albumin (Cys34 - the major site of modification) through a method developed in 2011 [23] that has been adapted for higher-throughput use in EXPOsOMICS adductomics studies [24]. The half-life of albumin (20–25 days) means that measurements of its covalent adducts reflect a longer “capture period” of exposure than other more transient omics biomarkers. The majority of studies to date have been concerned with quality control and methodological development and validation, but the first analyses in some of the EXPOsOMICS studies (PISCINA2, PEM, Oxford Street) and other epidemiological studies (i.e. EPIC) are starting to yield promising results with the identification of correlations between specific adducts and different environmental exposures or disease states. Further methodological developments are still required, including the development of new analytical methods and the creation of adduct libraries for annotation, but adductomics is set to become another key component in the study of the exposome.

### Statistics in Exposome Research

Marc Chadeau-Hyam and Roel Vermeulen presented “Statistics in Exposome Research: from omics profiling to dynamic modelling”. The presentation outlined some of the main challenges in the analysis of large, complex datasets produced by untargeted omics analyses in studies of the exposome (i.e. simultaneous testing of multiple hypotheses, consideration of multiple correlated exposures, of exposure interactions and non-linear exposure-response relations, and of temporal factors in exposures) together with the statistical tools being developed to address them [25]. Examples of the application of established statistical methods to analyses of data from EXPOsOMICS and HELIX studies were presented, including: a comparison of several multivariate regression-based methods for identifying true exposure-outcome associations from a large set of correlated exposures [26]; the use of techniques for analysing multivariate data, such as multi-level partial least squares (PLS) methods, in the analysis of data from the PISCINA2 study, for identifying specific molecular signatures representative of disinfection by-product exposure; the use of network representation methods for the identification of key signals or combinations of signals in omics data that play a pivotal role in describing the association between exposure and effect, illustrated by analyses of transcriptomic profiles in the PISCINA2 study and of epigenome-wide association studies of smoking and lung cancer risk [27]. Finally, the dissemination and training activities in statistical analysis of omics data integrated in the EXPOsOMICS and HELIX projects were also presented.

## Plenary discussion – External Exposome

The plenary session on the “External Exposome” led by Roel Vermeulen and Gary Miller discussed the following questions:

- What can be the contribution of exposure science to hazard identification and risk assessment?
- What is the state of the art of new exposure measurement technologies?
- What are the research needs?

The development of mobile technologies and of smaller, cheaper sensors allows for easier and more frequent collection of data on exposures in research participants (i.e. location, air pollutants, noise, diet) and the modelling of exposures in the wider population to numerous environmental factors [28]. Commercial personal monitoring devices will continue to get cheaper and more accurate and in a few years will likely be accurate enough for research purposes. There is also the potential for crowdsourcing data and the use of data from commercial providers, including social media, to capture a range of exposure data, including on the wider social-ecological context [29]. With better understanding of the correlation structure of many exposures, i.e. through correlation globes, we may in due time be able to construct Exposome Maps with smaller numbers of exposures being representative of a broader exposure profile (akin to HapMap) [30, 31].

There remain however a number of methodological challenges to document the external exposome (Table 2). There are currently many poor quality sensors on the market and a need for thorough validation of new and existing sensors. It would be useful to provide information on the quality of available devices such as the U.S. Environmental Protection Agency air sensors toolbox (www.epa.gov/air-sensor-toolbox) that provides general guidance to the public, researchers, and developers on available air pollution monitoring devices. It would also be useful to compare information obtained using different mobile applications such as those developed specifically for research purposes vs. commercially available apps (i.e. ExpoApp developed for the European CITI-SENSE project (http://www.citi-sense.eu/) [32] vs. commercially available Moves (https://moves-app.com/)). Although photo-based methods and wearable cameras have been implemented in epidemiological studies to better understand participant diet, location, or time-activity behavior patterns, further work is needed in the processing of such data. For example, in the case of dietary assessment to go beyond estimating food volume to recognising specific types of food or cooking method [33]. There is a need to define the optimal vs. sufficient level of resolution required for research and policy-making as well as participant adherence and measurement in large-scale populations. There are also outstanding questions regarding who will collect and store data as well as have access and ownership [28].

**Table 2:** Key scientific and policy challenges identified as part of the EXPOsOMICS project.

Type of Challenge	Challenge
Scientific	-Availability of accurate and inexpensive sensors -Low statistical power (low levels of exposure and small omic changes) -Multi-city designs are needed for contrast, but complicate inference -Validation in larger, longer-term exposed populations and in different settings and across omics platforms -Collection and storage of data as well as data access and ownership -Limited understanding of biological pathways and their interaction -Limited capacity to look at historical exposures -Large variation in bioinformatics analyses -Validation of omics approaches (both technical validation and biological validation) and a lack of a platform for data sharing -Matching omics measurements with functionalities (annotation of unknown biomarkers, differentiating exposures from biological responses, investigation of mixtures and interactions between agents) -Understanding mechanisms for biological plausibility and causality assessment -Cost of omics
Policy	-Current regulatory standards and policy not yet adapted to the use of EXPOsOMICS evidence -Conceptual framework for integrating the contribution of exposome or external exposure data into models of disease -Communication of research results -Demonstrate the utility of the exposome approach to funders and policy makers

## Plenary discussion – Internal Exposome

The plenary session on the “Internal Exposome” led by Paolo Vineis and Tim Gant discussed the following questions:

- What can be the contribution of omic measurements in hazard identification and risk assessment?
- What are the current limitations?
- What are the most urgent needs in the field of omics research?

At present omics approaches are useful in biomarker discovery and research, also for hypothesis generation, but it will take time to validate omics approaches to the point that there is sufficient confidence for use in regulatory and policy decisions. There are both *agnostic* and targeted approaches with different advantages (hypothesis generation vs. refining knowledge on mechanisms) and the opportunity for cross-validation and discovery of new biomarkers/mechanisms by combining both approaches. Omics data may also be useful to inform physiologically-based pharmacokinetic modelling (PBPK) to improve internal exposure estimates integrating epidemiology and toxicology approaches or as a tool to develop more cost effective interventions by focusing on the most relevant pathways/networks for prevention.

Limitations of omics are different depending on intended use, i.e. quantifying exposures or downstream markers of pathways associated with disease outcomes (Table 2). There is also a limited understanding of biological pathways, particularly interactions between different pathways. Although omics have contributed to advancing the field, i.e. use of overlaps across different omics to identify the most robust findings, there is a need to recognise the danger of over-interpreting results. There is a limited capacity to look at historical exposures - adductomics may provide longer-term information compared to other omics, but this still requires validation. There is difficulty in distinguishing effects of exposures from the effects of disease processes associated with them in analyses of the internal exposome. There is also a large variation in bioinformatics analyses of omics data and consequently a need for increased standardization and reproducibility. There is a need for validation of omics approaches (both technical validation and biological validation) and a lack of a platform for data sharing. A recommendation is to develop an international initiative to promote data sharing and setting of standards for the reporting and validation of omics markers. Finally, although the cost of omics analysis is decreasing, it remains a limiting factor in most studies.

## Plenary discussion: Policy translation

The plenary session on “Policy translation” led by David Balshaw and Christopher Wild discussed the following questions:

- In the light of the philosophy expressed in the U.S. National Academy of Sciences (NAS) report on pathway perturbation, what is the potential contribution of the exposome paradigm?
- How does it fit into the strategies of environmental and public health agencies, NGOs, regulatory agencies, industry and academia? What institutional actors are necessary?
- How should research be funded to meet the next challenges of exposome research?

Before discussing the potential contribution of the exposome to policy development it was important to consider what is meant by policy: guidance, recommendations and legislation each has different requirement levels for evidence. It is also important to bear in mind the many factors that affect the translation of science into policy including public and media pressure, economic interests, and political agendas, for example.

On a basic level, exposome research can be seen as replicating the approaches of classic risk assessment with higher resolution and greater accuracy. This includes improved exposure assessment with the ability to capture correlated co-exposures, complex mixtures, and synergies, the provision of dose-response data including at low-dose exposures, and biological plausibility of exposure-disease associations by bridging experimental and human data (for example by identifying the same epigenetic or metabolomic signatures in both animal and human studies). Identifying susceptible sub-groups and critical windows of exposure, monitoring prevalence and level of exposure and evaluating interventions through short-term endpoints and/or mechanism-based markers can also be performed.

However, pathway perturbation (a more general concept than the adverse outcome pathway (AOP)) is a change of paradigm, a new way of thinking about hazard identification and risk assessment by using pathway analysis to link multifactorial causality with risk decisions [34]. Exposome research can provide essential information on early perturbation of pathways at low levels of exposure, as this project shows in relation to air pollution and water contaminants. The possibility of evaluating complex mixtures and synergies between compounds is also a change of paradigm from evaluating risk for individual agents. Our understanding of the dynamic changes and interactions in pathways and the way they relate to exposures are still patchy; this limits the way pathway analysis can be used to identify multifactorial aetiology underlying disease, at least at present. Further, current regulatory standards and policy currently focus on animal models for mechanistic evidence and biological plausibility and epidemiological evidence for strength of association as pre-requisites (i.e. Bradford-Hill causality assessment criteria [35]) which are also not yet adapted to the use of EXPOsOMICS evidence (pathway analysis/pathway perturbation) for risk assessment.

There is an opportunity for EXPOsOMICS to contribute to breaking the institutional silos in policy-making organisations, by promoting integrated approaches that examine the effects of multiple categories of agents in a more holistic approach to risk assessment [36]. However, policy development is typically slow due to powerful pressures from existing interests; this will condition the speed with which novel approaches and data from EXPOsOMICS will be accepted for translation into policy. In the face of resistance from vested interests, translating evidence into policy requires strong, well organised commitment; including the consideration of engaging with other groups in society with an interest in the protection of public health and the environment either as a direct or co-benefit.

Considerations for exposome research to better link with policy-making were also discussed including noting the big gap in the way questions are framed in a scientific vs. a regulatory/policy context and a need to consider from the stage of design how study results can be relevant to and presented in a way that can be integrated into regulatory/decision-making processes (Table 2). Improved dialogue with policy-makers is needed to better understand research needs for policy-making and in the translation of EXPOsOMICS findings into understandable messaging.

## Complementarities with other initiatives

### Helix

Martine Vrijheid presented an overview of the progress of the HELIX project [37] www.projecthelix.eu and complementarities with EXPOsOMICS. HELIX provides a wide coverage of the exposome during the early-life, including a range of 200–300 individual and chemical (i.e. polychlorinated biphenyls, phthalates, metals, social factors), outdoor urban (i.e. air pollutants, noise, built environment/green spaces), and internal environments (i.e. metabolomics, proteomics, transcriptomics, DNA methylation), based in six existing European birth cohorts. The main progress relates to describing the exposome and its determinants across Europe in mothers and their children, including correlations between exposures [31]; characterizing personal exposomes and short- and long-term variability both within and between participants; determining omics signatures related to multiple early life environmental exposures; and relating the exposome to child health. HELIX contributes comparable biomonitoring, geospatial, and omics data in several European countries which can be used to identify high-risk groups and provide a holistic picture on important sources and determinants of multiple environmental exposures. It provides a toolkit for personal exposome assessment including various personal-level sensors, an in-depth characterisation of temporal variability in exposures through repeated sampling, and molecular fingerprints within and between individuals [38]. Investigation of omics signatures and molecular pathways in vulnerable time periods can be used for improved risk assessment and prediction of future disease risk as well as better understanding of biological mechanisms when combined with pathway analysis. Finally, systematic evaluation of child health effects of multiple exposures allows for the identification and prioritization of important environmental exposures, and the estimation of their associated health impact to ultimately lead to improved prevention strategies.

### NIH/NIEHS

David Balshaw outlined “Exposome initiatives at NIH/NIEHS” including current approaches in exposome research in light of the recent U.S. NAS report [34], and the parallels to the EXPOsOMICS and HELIX programmes. The exposome concept provides a framework for moving environmental health research from a reductionist approach – one exposure, one disease – to considering the influence of multiple stressors at multiple time-points. Research on the exposome is moving from concept to the demonstration stage – starting to show with the limited tools available at present some of the potential of this approach, but requiring in parallel the development of capacity to look at multiple exposures through the development of better technical and methodological tools and validation in real-world conditions. The Children’s Health Exposure Analysis Resource (CHEAR) study was presented as a demonstration case for exposome research at NIEHS, comprising two main components, research and development of infrastructure. The early stages have focused on development of standards and quality assurance to improve reproducibility of the analyses, and the creation of a data repository for health histories and omics data. The potential for cross-validation between targeted (hypothesis-driven) and untargeted (agnostic) approaches and the use of additional data on biological responses to anchor targeted and untargeted analyses are being evaluated. Future perspectives include exploring the contribution of the exposome and multi-omic integration for mechanistic research and systems biology. Some of the key immediate challenges in exposome research include: compound identification from untargeted analyses; making data accessible for further analyses; and using the exposome concept as a tool for prevention.

### US NAS Twenty-First Century Risk Assessment

Jonathan Samet presented the report “Using 21st Century Science to Improve Risk-Related Evaluations” developed by a committee convened in 2016 by the U.S. NAS [34]. The report builds on earlier initiatives in the U.S. (“Toxicity testing in the 21^st^ Century” in 2007 [39] and “Exposure Science in the 21^st^ Century” in 2012 [40]) and internationally (REACH programme in Europe), to guide the development of new scientific and technical methodologies for exposure monitoring, toxicological evaluation, epidemiology, and their application to risk assessment. Advances in exposure science, with the increasing use of more sophisticated exposure monitors and the use of omics technologies to investigate biological responses, present new opportunities for example for the analysis of multiple exposures and of pathways linking exposures and outcomes, however they also present challenges in the analysis, integration and interpretation of large volumes of diverse data, and on linking these to potential risks for human health. The concepts of “pathway perturbation” and “meet-in-the-middle”, i.e. identifying biomarkers linking exposures and disease outcomes, were identified by the NAS committee as central to these new approaches for risk assessment. Finally, the criteria traditionally used for causal assessment, such as the Bradford-Hill criteria (also above), will need to be developed and adapted to integrate these new approaches, and for the moment guided expert judgement should be used for integrating the diverse data streams to draw causal conclusions.

## The future of the Exposome

### The New Science of Exposure Assessment

Mark Nieuwenhuijsen, in a presentation “The new science of exposure assessment”, highlighted new developments in assessments of the outdoor exposome, including in environmental measurements and modelling, and remote and personal sensing. Challenges in this area include: improving resolution of remote sensing and the accuracy and miniaturisation of personal sensing devices [32]; increasing accessibility of environmental data through the creation of data repositories; and integrating different sources of information, i.e. remote sensing, ambient monitoring, modelling and personal monitoring [41]. The remarks by Gary Miller as the discussant, and the discussion that followed, emphasised the importance of the complementarity of the approaches provided by the external and internal exposome studies, not just by increasing confidence in matching observations, but also by making the direct link between pathway perturbations observed in studies of the internal exposome, with external factors that can then be identified as priorities for preventive interventions and policy making. The speed of technological developments in this area presents good opportunities but limited funding continues to be a major challenge and delays further significant advances.

### Weak Carcinogens and “Pathway Perturbation”

Paolo Vineis in a presentation “Weak carcinogens and “pathway perturbation””, highlighted some of the ways in which EXPOsOMICS research is contributing to assessments of carcinogenicity, including: contributing mechanistic data to biological plausibility assessments; contributing to the refinement of models of carcinogenesis (hallmarks of carcinogenesis [42, 43]), although still missing is a conceptual framework for integrating the contribution of exposome or external exposure data into these models; contributions to improved measurements and identification of omics alterations at low doses; in conjunction with epidemiological studies contributing additional data to causality assessment for weak carcinogens. Tim Gant, as a discussant, emphasised the different perspectives from epidemiology and toxicology on carcinogen identification, and highlighted some of the gaps in our current understanding of the downstream effects of observed pathway changes, including: difficulty in distinguishing between transient changes in response to a short-term stressor (homeostasis) and long-term changes in response to a chronic exposure; difficulty in distinguishing between pathway changes that lead to an adverse effect vs. those that lead to metabolic adaptation to a low-dose exposure (hormesis). There is a difficulty in funding work on developing the technical resolution to address these gaps in knowledge, but it is increasingly needed. In the discussion that followed the use of the term “weak carcinogens” was challenged: the meaning is difficult to define (weak association? weak evidence?) and problematic as it is open to misinterpretation in policy settings as being unimportant and so easily dismissed.

### New Developments of the Exposome Concept

Lastly, Christopher Wild presented on “New developments of the “Exposome” concept”. The presentation highlighted the remarkable progress in exposomics research in a short period of time [44–48]. Being an emerging discipline, our understanding still necessarily has gaps but we should not be discouraged by these limitations as long as we recognise limitations in the interpretation of the data. Major challenges for exposome research remain: validating exposure measurements (reliability of individual measures) [49], data integration and analysis (understanding correlations of exposures and the role of confounders) [47], matching omics measurements with functionalities (annotation of unknown biomarkers; differentiating exposures from biological responses; investigation of mixtures and interactions between agents; understanding mechanisms for biological plausibility and causality assessment). It is important to remain focused on the key questions – characterizing exposure-disease relationships – and to invest further in method development and validation. The discussant Marco Martuzzi, emphasised the importance and relevance of research on the exposome in the context of the development of interventions targeted at the population level to improve public health [50, 51]. In contrast, the application of the genome lies in interventions targeted to individuals. The need to develop criteria for defining environmental health priorities to guide priority setting and investment in research informing policy development was also highlighted.

## Conclusion

The meeting concluded with feedback on the EXPOsOMICS project from the European Commission and the International Scientific Advisory Board. Overall, EXPOsOMICS provides proof-of-principle that an exposome approach can lead to important findings that have an impact both on knowledge of the mechanisms linking exposure to common pollutants with diseases, and on preventive and regulatory action. The potential of exposome research to contribute to policy development includes: improved exposure assessment; enhanced specificity of actions to remove environmental hazards; identification of subgroups at risk; enhanced prediction and prevention of disease by early intervention; monitoring of results of policies in reducing exposures; and elucidation of new hypotheses on the role of environment and health. Current limitations of this area were discussed in terms of showing added value for public health including the need to: improve communication of research results to non-scientific audiences and promote interaction among the producers and users of research; target research to the broader landscape of societal challenges – i.e. target priority policy areas that are under-researched or where current methods do not provide appropriate answers; and become better at drawing applicable conclusions – i.e. what is the added value of the research and what is the follow-up. Priorities for future work include the development and standardization of methodologies and technologies for assessing the external and internal exposome, improved data sharing and integration, and the demonstration of the added value of exposome science over conventional approaches in answering priority policy questions. Being a new field, there will be a need to continue to demonstrate the utility of the exposome approach to funders and policy makers.

## Abbreviations

AOP: Adverse outcome pathway
CC16: Club cell protein
CHEAR: Children’s Health Exposure Analysis Resource
CVD: Cardiovascular disease
DBPs: Disinfection by-products
IHD: Ischemic heart disease
LUR: Land use regression
NAS: National Academy of Sciences
NO_2_: Nitrogen dioxide
PBPK: Physiologically-based pharmacokinetic modelling
PEM: Personal exposure measurements
PLS: Partial least squares
PM_10_: Particulate matter less than 10 μm in diameter
PM_2.5_: Particulate matter less than 2.5 μm in diameter
RR: Relative risk
THM: Trihalomethanes
UFP: Ultrafine particles
